# Parkinsonism Hyperpyrexia Syndrome: A Rare Cause of Temperature Elevation

**DOI:** 10.7759/cureus.79801

**Published:** 2025-02-27

**Authors:** Maria Margarida Rosado, Sara Campos, Nuno Bernardino Vieira

**Affiliations:** 1 Internal Medicine, Unidade Local de Saúde do Algarve - Hospital de Portimão, Portimão, PRT

**Keywords:** bromocriptine, drug-induced fever, fever, hyperthermia, parkinsonism hyperpyrexia, parkinson's disease

## Abstract

Fever is a common finding in hospitalized patients, and a noninfectious cause is present in up to half of cases. Parkinsonism hyperpyrexia syndrome is a rare and potentially fatal complication of Parkinson's disease, characterized by the presence of clinical features of neuroleptic malignant syndrome in patients who have reduced or completely discontinued antiparkinsonian medications. We report the case of a 63-year-old female patient with Parkinson's disease presenting with a sensorium alteration and noncompliance with her usual medication, parkinsonism, rhabdomyolysis, acute kidney injury, and transaminase elevation. Due to the persistence of the fever, despite resuming her usual antiparkinsonian medications, bromocriptine was initiated with a resolution of symptoms. We conclude that a thorough clinical history, with a drug compliance review, should be made to ensure a correct diagnosis and prompt treatment initiation.

## Introduction

Fever is a common finding in hospitalized patients. A noninfectious cause of fever is present in up to half of cases, and when an infectious cause is excluded, the diagnosis may not be made in over 90% of cases [1]. While the mortality from a noninfectious cause is lower, it remains significant, and unlike in sepsis, a higher temperature is associated with a worse outcome [2].

Noninfectious causes to consider in the differential diagnosis of fever include drug-related fever, inflammatory diseases, malignancy, endocrine and metabolic causes, thromboembolic disease, and central fever [3].

While fever is an elevation of body temperature that exceeds the normal daily variation, induced by cytokine activation and with an increase in the hypothalamic set point, hyperthermia is an elevation in core body temperature due to thermoregulation failure, not involving pyrogenic molecules, with an uncontrolled increase in body temperature that exceeds the body's ability to lose heat [4]. Hyperthermia can be induced by exercise, due to inadequate means of heat dissipation, or due to impairments of pathological or pharmacological thermoregulatory mechanisms, usually induced by drugs [5]. The main causes of drug-induced hyperthermia include malignant hyperthermia, neuroleptic malignant syndrome, anticholinergic syndrome, and sympathomimetic syndrome [3].

Parkinsonism hyperpyrexia syndrome is a rare and potentially fatal complication of Parkinson's disease, characterized by the presence of clinical features of neuroleptic malignant syndrome in patients that rapidly reduce or completely discontinue antiparkinsonian medications [6].

## Case presentation

A previously independent 63-year-old woman with Parkinson's disease (*PARK2* gene mutation), depression, and cluster B personality disorder presented to the Emergency Department (ED) with a one-day history of behavioral alteration, with drowsiness alternating with hypervigilance, having sustained a fall from her own height without loss of consciousness or head injury. She was usually followed by Neurology and was medicated with levodopa + benserazide 50 + 12.5 mg 3id, amantadine 100 mg 2id, rotigotine 8 mg id, quetiapine LP 150 mg 2id, quetiapine 25 mg 1id, and diazepam 2.5 mg 2id. Due to the sensorium alteration, she was non-compliant with medication for the previous 24 hours, with abrupt cessation of her usual medication.

The physical examination was remarkable for drowsiness, time and space disorientation, dysphonia, generalized tremor, predominantly orofacial and in the arms, myoclonus in the arms, and generalized hypertonia. During the ED stay, due to severe psychomotor agitation, intramuscular haloperidol 5 mg and chlorpromazine 25 mg were administered. One day after the ED admission, she developed a fever, with a tympanic temperature of 39.1ºC, and was admitted for further investigation.

Initial blood tests were remarkable for minor rhabdomyolysis (total creatine kinase 712 U/L), acute kidney injury (creatinine 1.56 mg/dL), hypernatremia (sodium 149 mmol/L), and minor elevation of transaminases, without elevation of inflammatory markers or other relevant alterations (Table 1).

**Table 1:** Relevant initial blood tests

Parameter	Result	Reference Value
Erythrocytes	3.72×10^6^/µL	4.00-4.80
Hemoglobin	11.5 g/dL	12.5-16.0
Hematocrit	34.0%	37.0-47.0
Mean corpuscular volume	91.3 fL	78.0-100.0
Mean corpuscular hemoglobin	31.0 pg	27.0-33.0
Leukocytes	11.9×10^3^/µL	4.0-10.0
Neutrophils	5.530×10^3^/µL	2.0-8.0
Lymphocytes	5.330×10^3^/µL	1.0-5.0
Monocytes	0.870×10^3^/µL	0.1-1-0
Eosinophils	0.050×10^3^/µL	0.0-0.8
Basophils	0.120×10^3^/µL	0.0-0.2
Platelets	268×10^3^/µL	150-400
Glucose	197 mg/dL	70-105
Urea	105 mg/dL	16-46
Creatinine	1.56 mg/dL	0.50-0.90
Sodium	149.0 mmol/L	136.0-145.0
Potassium	3.93 mmol/L	3.50-5.10
Chloride	110.6 mmol/L	98.0-107.0
Total calcium	10.0 mg/dL	8.6-10.2
Magnesium	2.70 mg/dL	1.45-2.67
Total bilirubin	0.5 mg/dL	0.2-1.0
Aspartate transaminase	53 U/L	10-30
Alanine transaminase	38 U/L	10-36
Gamma-glutamyltransferase	11 U/L	5-36
Alkaline phosphatase	114 U/L	0-120
Lactate dehydrogenase	333 U/L	135-214
Total creatine kinase	712 U/L	20-180
C-reactive protein	3.6 mg/L	0.0-5.0
Albumin	4.4 g/dL	3.5-5.0

**Figure 1:**
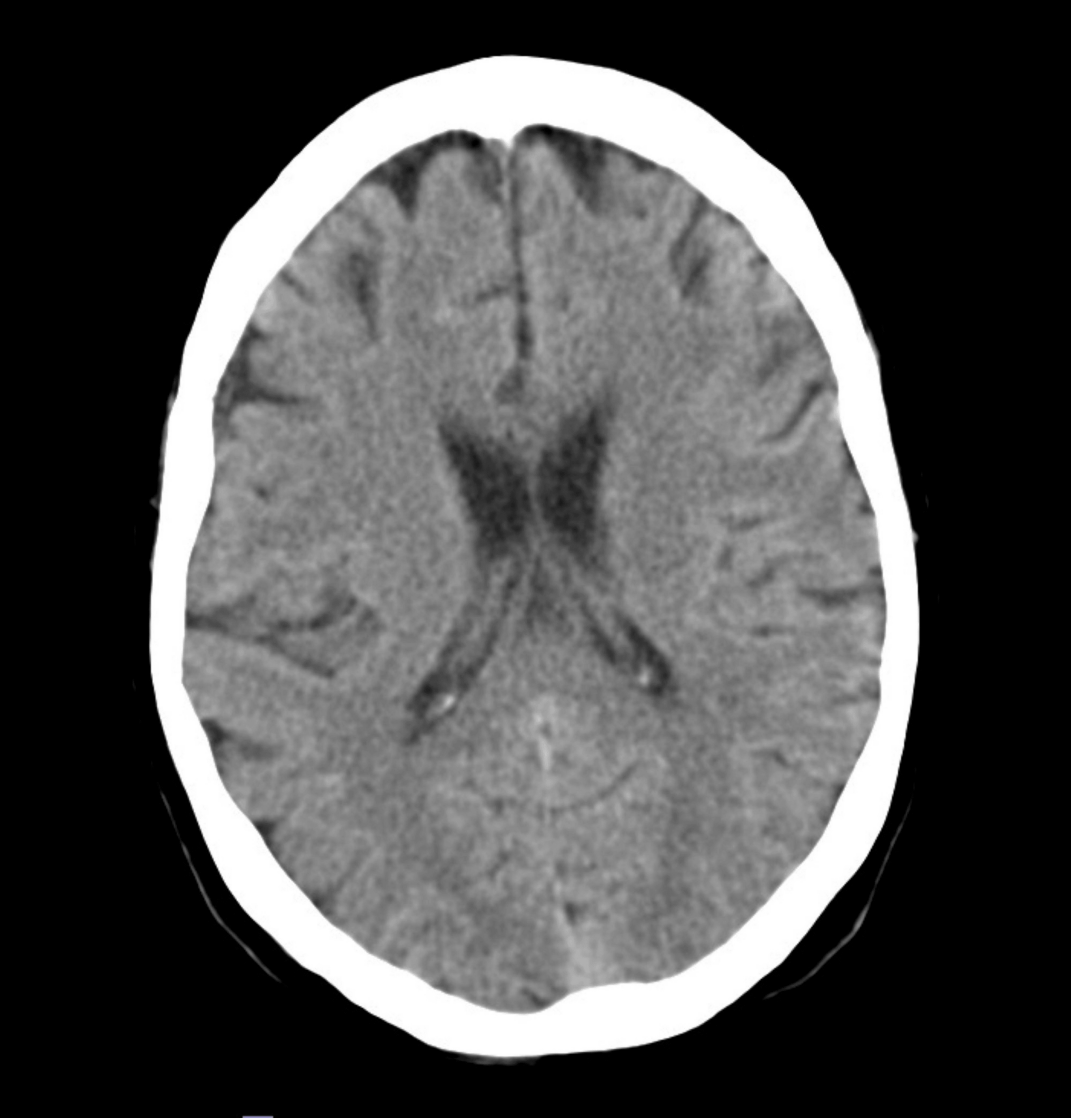
Head computed tomography without acute alterations

A head computed tomography was performed, revealing no acute alterations (Figure 1). Procalcitonin was negative (0.12 ng/mL). Urine and blood cultures were collected, yielding negative results, and a lumbar puncture was performed, revealing unremarkable cerebral spinal fluid (Table 2).

**Table 2:** Cerebrospinal fluid findings

Cerebrospinal Fluid Parameter	Result	Reference Value
Glucose	121 mg/dL	50-80
Total protein	47 mg/dL	15-50
Cell count	1 cell/mm^3^	<10
Direct exam (Gram stain)	Negative	-
Culture	Negative	-

After the exclusion of an infectious cause and due to the suspicion of drugs contributing to the temperature elevation, her usual antiparkinsonian medications were reinitiated, neuroleptic medications were suspended, and the benzodiazepine dose was increased. Despite this, the patient maintained a fever, with a tympanic temperature of 38.5-39.5ºC, not responding to antipyretic medications, and developed hypertension and dysphagia.

**Figure 2:**
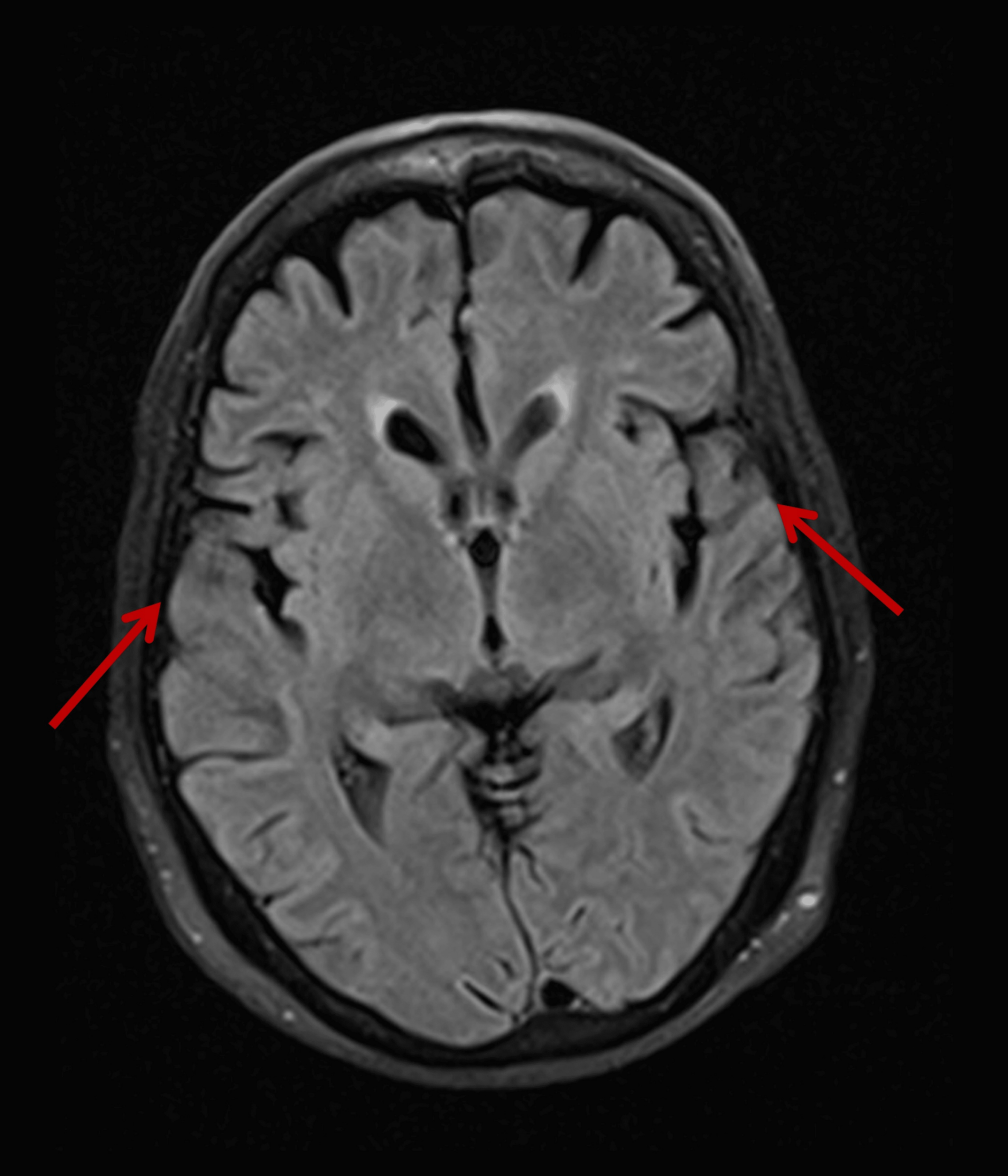
Brain MRI showing temporo-insular alterations compatible with Parkinson's disease (arrows)

Brain magnetic resonance imaging (MRI) was performed (Figure 2), showing temporo-insular alterations secondary to Parkinson's disease, without other relevant findings.

Due to the persistence of pyrexia, bromocriptine 2.5 mg every six hours was initiated, and the amantadine dosage was increased to 100 mg every eight hours, with a posterior increase of the bromocriptine dosage to 5 mg every eight hours. After five days, fever, extrapyramidal signs, and an altered state of consciousness completely resolved. Bromocriptine was maintained for four weeks, and after its discontinuation, the patient remained well.

## Discussion

Parkinson's disease is a multisystemic neurologic disorder characterized by the loss of dopaminergic neurons, and pharmacologic therapy to control motor symptoms includes dopaminergic and nondopaminergic medications [7].

Parkinsonism hyperpyrexia syndrome occurs in patients with Parkinson's disease after an abrupt change in dopaminergic medication, with an incidence of 0.3% [8]. Its clinical features are similar to those of neuroleptic malignant syndrome due to a similar pathophysiology, with sudden central dopaminergic hypoactivity and peripheral sympathetic hyperactivity [6].

Typically, symptoms manifest within 18 hours to seven days after a change in dopaminergic medications, characterized by worsening parkinsonism, marked hypertonia, an increase in body temperature, altered consciousness, and dysautonomia [9]. Symptoms usually occur in succession, with motor symptoms followed by hyperthermia, altered consciousness (ranging from confusion to coma), dysautonomia (tachycardia, labile blood pressure, diaphoresis, functional ileus), and lastly, dysphonia and dysphagia [6]. Blood tests may reveal leukocytosis, elevated creatine kinase (CK) levels, altered liver enzymes, acute kidney injury, metabolic acidosis, and disseminated intravascular coagulation [10].

Despite the similarity to neuroleptic malignant syndrome, some distinguishing features exist: the baseline disease is different, the precipitating factor is distinct, there may be a longer latency until symptom development, parkinsonism is more evident, CK elevation and leukocytosis may be less pronounced, and the prognosis is more favorable, with a mortality of only 4% [6,8].

Parkinsonism hyperpyrexia syndrome is a neurological emergency, and early diagnosis and prompt treatment are essential. In addition to supporting measures that include external cooling and intravenous hydration, prompt reintroduction of antiparkinsonian medications is vital, with levodopa in the same dosage as previously taken being the first line treatment [10]. If up-titration of levodopa is ineffective, other dopamine agonists (oral ropinirole or pramipexole, transdermal rotigotine, subcutaneous apomorphine, or oral or intravenous amantadine) can be used [8]. Oral bromocriptine and dantrolene are also frequently used [9,11]. Early withdrawal of treatment with bromocriptine and dantrolene may cause rebound symptoms, and it is therefore recommended to continue treatment for approximately 10 days after the resolution of symptoms [8].

In the case presented, the patient had two predisposing risk factors (sudden discontinuation of antiparkinsonian medications and the use of antidopaminergic medications), displayed the typical clinical triad (hyperthermia, extrapyramidal symptoms, and altered consciousness), and showed improvement and resolution of symptoms after reintroduction and titration of dopaminergic medications, confirming the diagnosis of parkinsonism hyperpyrexia syndrome.

## Conclusions

Parkinsonism hyperpyrexia syndrome is a rare, life-threatening disorder that can easily be misdiagnosed. This case demonstrates the importance of patient and caregiver awareness of the risk of abrupt discontinuation of antiparkinsonian medications. For any patient with Parkinson's disease with acute mental status deterioration of unknown etiology, a high index of suspicion for parkinsonism hyperpyrexia syndrome should be present. A careful and thorough clinical history, with drug and drug compliance reviews, should be made to ensure a correct diagnosis in most cases. Prompt treatment should be initiated and includes supportive therapy and dopaminergic medications with appropriate up-titration until the resolution of symptoms.
